# Screening of Zoonotic Parasites in Playground Sandboxes of Public Parks from Subtropical Mexico

**DOI:** 10.1155/2019/7409076

**Published:** 2019-06-02

**Authors:** Gonzalo A. Pacheco-Ortega, José I. Chan-Pérez, Antonio Ortega-Pacheco, Eugenia Guzmán-Marín, Melissa Edwards, Mark A. Brown, Matilde Jiménez-Coello, Ivonne B. Hernández-Cortazar

**Affiliations:** ^1^Laboratorio de Biología Celular, Centro de Investigaciones Regionales “Dr. Hideyo Noguchi”, Universidad Autónoma de Yucatán, 97000 Mérida, Mexico; ^2^Depto. de Medicina Interna y Cirugía. Campus de Ciencias Biológicas y Agropecuarias, Universidad Autónoma de Yucatán, 97100 Mérida, Mexico; ^3^Office for Undergraduate Research and Artistry, The Institute for Learning and Teaching, Colorado State University, Fort Collins, CO 80521, USA

## Abstract

The pathological agents* Toxoplasma gondii, Ancylostoma caninum, *and* Toxocara canis *are widely distributed zoonotic parasites with high prevalence in tropical and subtropical regions of the world. The aim of the present study was to determine the presence of DNA from these parasites in sand samples from the sand playgrounds in the southeastern region of Mexico. Samples of sand were collected from 68 playgrounds in public parks in the city of Merida, Yucatan, which is the main urban area in the southeast of Mexico. The samples were examined using nested PCR to detect the SAG1 gene from* Toxoplasma gondii*, and endpoint PCR for the amplification of ITS-2 and rRNA-ITS2 genes from* Toxocara canis* and* Ancylostoma caninum,* respectively. The presence of* T. gondii *DNA was detected in 11.8% (8/68) samples, DNA from* A. caninum* and* T. canis* was not detected. Results indicate that playgrounds from the studied sandboxes are contaminated with* T. gondii* oocysts and may represent a risk of infection for people in contact with the sand, especially for preschoolers.

## 1. Introduction

Parasitic zoonoses transmitted by cats and dogs represent a serious problem of public health. The overpopulation of stray dogs and cats which exists predominantly in metropolitan areas increase the risk of contamination of public spaces with infected stools. Among the principal zoonotic parasites transmitted by cats,* Toxoplasma gondii *is one of the most commonly reported and* Toxocara canis* and* Ancylostoma caninum *are common in dogs, particularly when free roaming and without the basic preventive medicine [1, 2].

The obligate intracellular protozoan* T. gondii* has a worldwide distribution and is capable to infect humans and all warm-blooded animals including mammals and birds. It is estimated that one-third of the world population have been exposed to* T. gondii *[3], and the highest prevalence have been reported in countries from Latin America and African tropical countries [4]. Felids are the key animal species in the life cycle of* T. gondii* because they are the hosts which allows the completion of sexual reproduction in their gut and later are able to shed the environmentally resistant stage, the oocyst [5], after a primary infection felids are capable of shedding up to 10 million oocysts in just one day and usually only shed the organism for a short period of time [6]. On the other hand,* T. gondii* oocysts are highly resistant to warm and humid conditions, staying viable in soil for up to 21.5 months [7]. This highlights the risk that they represent to acquire the infection. The majority of horizontal transmissions to humans are caused either by the ingestion of tissue cysts in undercooked infected meat or by the ingestion of water, food, or soil contaminated with sporulated oocysts derived from feline feces [8]. In Europe, it has been reported that the ingestion of sporulated oocysts represents 6-17% of infections in pregnant women [9], constituting an important way of transmission. The presence of* T. gondii* oocysts in environmental samples has been reported in China and France with positive results in soil samples of 12.69 and 29.2%, respectively [10, 11]. A study carried out in Brazil isolated DNA of* T. gondii* in 25.8% of 31 samples obtained from playgrounds of elementary public schools [12]. Numerous outbreaks of symptomatic toxoplasmosis related to the ingestion of oocysts from soil contamination have been reported [13–15].

*Toxocara canis* is an ascarid nematode with a worldwide distribution; their definitive hosts are the domestic dog and cat. Infection occurs when the definitive host and the intermediate host (humans) ingest the embryonated eggs of the parasite from contaminated sources (e.g., soil); this contamination is the result of the indiscriminate defecation by the definitive hosts. These eggs can remain viable for months to years outside of the host due to a resistant outer shell composed of ascarosides; this layer enables eggs to withstand various harsh chemicals, extreme temperature changes, and various degrees of moisture [16]. It has been recognized that the use of public parks is an important risk factor for the acquisition of* T. canis* infections in children [17]. The prevalence of infection in humans is variable; it tends to be smaller in industrialized countries (0.7-2.4%) when compared with less industrialized ones (63.2-92.8%) [18]. In Mexico, studies have reported a prevalence of 12.02 to 22.2% in children [19, 20]. Also, studies have been conducted for the detection of eggs from the parasite in public parks from Portugal, Poland, Ireland, and Mexico among others, with a prevalence of 85.7%, 53%, 15%, and 60%, respectively [21–24].

*Ancylostoma caninum* is also a nematode parasite with the dog being its definitive host. Infection occurs when the host eliminates eggs through defecation; 5 to 10 days later, they transform into their infective stage of filariform larvae which can invade humans via hair follicles and trough small fissures on skin until they reach the small bowel, their definitive habitat. In humans, this can cause an eosinophilic gastroenteritis and chronic iron deficiency anemia which can result in long-term poor health outcomes like reduced cognitive, intellectual, and physical development and reduced fertility among women [25, 26]. Environmental contamination has been evaluated in multiple countries and tends to be higher in tropical regions. Argentina, Brazil, and Venezuela reported prevalence rates of 20.5, 64.8, and 61.1%, respectively, while countries like Spain, Italy, and Poland reported much lower prevalence, 3, 7, and 3.2%, respectively [27].

The southern region of Mexico is considered an endemic area for these zoonoses where prevalence of infection in humans have been reported to be 90% for* T. gondii* and 29.2% for* T. canis *[28], and with regard to* A. caninum,* there are no available studies yet. However, studies evaluating environmental contamination with these parasites in this region are scarce. In a study conducted in a region of southern Mexico (Campeche city), the contamination with* A. caninum* of 92.8% of stools collected from 14 public parks was reported [29]. In the study region, the presence of* A. caninum* and* T. canis* eggs was reported in dog stool samples of 73.8 and 6.2%, respectively [30]. Only one study has been made in this region with regard to the environmental contamination with* T. gondii*, finding the presence of DNA in 5.4% of samples of public drinking water in an urban area [31]. No studies evaluating the presence of DNA nematodes in soil samples from public parks have been reported; instead, studies generally report the presence and abundance of eggs. Therefore, the aim of this study was to determine the presence of* T. gondii*,* T. canis,* and* A. caninum* DNA in sand samples of playground sandboxes from public parks in southeastern Mexico.

## 2. Materials and Methods

### 2.1. Study Area and Sampling

This study was conducted in the city of Merida, the capital of Yucatan, Mexico (19°30′ and 21°35′ N in latitude, and 87°30′ 90°24′ W in longitude). The climate in the region is tropical (Aw) with an average annual temperature of 24-28°C and a range of total annual rainfall of 400-2000 mm [32]. Samples were obtained during the month of July 2017 and during June-July 2018. A cross-sectional study was carried out by collecting sand samples from 68 playground sandboxes in public parks of Merida; from each park, 20 gr of sand were obtained, 10 gr from the superficial region (< 2 cm) and 10 gr from a deeper region (2-10 cm or until reaching rock bottom). Each sample was divided in two 5 gr samples, resulting in 4 subsamples from each park; samples were placed in sterile tubes of 50 mL.

### 2.2. Extraction and Purification of Sand Samples

Extraction was performed by following the methodology described by Lélu* et al*. [33]; briefly, to each 5 gr sample, 10 mL of deionized water was added; posteriorly, it was mixed for 1 minute using a vortex mixer. Then, 20 mL of Sheather's sugar solution (specific gravity: 1.2) was added and centrifuged at 1500 g for 20 minutes; the obtained interface (13 mL) was transferred to another tube in which 35 mL of deionized water was added and then centrifuged at 1500 g for 20 minutes. From each tube, 1 mL of sediment was collected and placed in a 1.7 mL Eppendorf tube; this was centrifuged at 1500 g for 5 minutes and, then, the supernatant was eliminated (approximately 600 *μ*L), and the remaining sediment from each vial was then combined in one tube, resulting in one tube for each sample (superficial and deep) from their respective parks. Purification was made by using the NucleoSpin® TriPrep (MACHERY-NAGEL, Germany) kit and following its manufacturer recommendations.

### 2.3. Nested PCR for the Detection of T. Gondii DNA

The nested PCR (nPCR) was used to amplify a fragment of 390 pb of SAG1 gene (main surface protein of* T. gondii*), using a thermocycler Veriti 96 wells (Applied Biosystems™). The first amplification was performed with the external primers sense 5′-GTTCTAACCACGCACCCTGAG-3′ and antisense 5′- AAGAGTGGGCTCTGTGA - 3′; in the second amplification, primers used were internal sense 5′- CAATGTGCACCTGTAGGAAGC-3′ and internal antisense 5′-GTGGTTCTCCGTCGGTGTGAG-3′ [34]. The first amplification reaction was performed with 1X PCR buffer (PROMEGA) at a concentration of 2mM of MgCl^2^; 0.8mM of dNTPs; 0.5 uM for each primer; 1.5U Taq polymerase; and 2 *μ*L of DNA sample in a final volume of 25 *μ*L. The second run had the same conditions as the first PCR; only the concentration of primers was 0.3 uM and for the second run 2 *μ*L PCR of the product from the first round was used. The PCR conditions in the first run were 95°C for 5 min, followed by 30 cycles at 94°C for 30 s, 55°C for 1 min, and 72°C for 2 min. In the second run, it was 95°C for 5 min, followed by 35 cycles at 94°C for 30 s, 60°C for 1 min, and 72°C for 1 min and 30 s. As a positive control, DNA from* T. gondii *tachyzoites (1x10^2^) of reference strain of* Toxoplasma gondii* (RH) strain was used; as a negative control, a master mix without DNA was used. The amplification products (390 pb) were visualized on agarose gel 1.5%, stained with ethidium bromide (0.5 *μ*g/mL) using a Gel Doc™ XR+ Gel Documentation System (Bio-Rad™).

### 2.4. PCR for the Detection of A. Caninum and T. Canis DNA

An endpoint PCR was used to amplify a fragment of 380 pb of ITS-2 gene from* Toxocara canis* with the primer sense Tcan1 5′AGTATGATGGGCGCGCCAAT-3′ and antisense NC2 5′-TTAGTTTCTTTTCCTCCGCT-3′ [35] and a fragment of 427 pb of rRNA-ITS2 gene from* Ancylostoma caninum* with the primer sense A.canF 5′-AGCATTAGGCTAACGCCCGA-3′ and antisense A.canR 5′-AACGAGTTTGCTGTCATTCAGTCC-3′ [36]. The conditions for the PCR reaction were buffer PCR GoTaq® Green 1X (PROMEGA) at a concentration of 3mM of MgCl^2^; 0.8 mM of dNTPs; 0.5 uM for both primers (forward and reverse); 1.5U Taq polymerase; and 3*μ*L of DNA sample in a final volume of 20 *μ*L. The PCR conditions were 94°C for 10 min, followed by 30 cycles at 94°C for 30 s, 60°C for 30s, and 72°C for 7 min, using a thermocycler Veriti 96 wells (Applied Biosystems™). As positive controls, DNA from* T. canis *adult nematodes and DNA from* A. caninum* eggs obtained from naturally infected dogs were used; as a negative control, a master mix without DNA was used. The amplification products were visualized on agarose gel 1.5%, stained with ethidium bromide (0.5 *μ*g/mL) using a Gel Doc™ XR+ Gel Documentation System (Bio-Rad™).

## 3. Results

**Figure 1:**
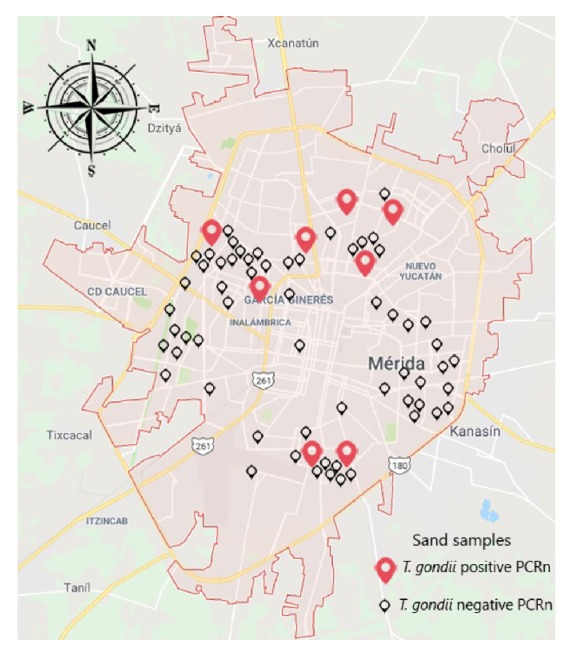
Distribution of sampled points of sand from sandbox playgrounds in the city of Merida, Yucatan, Mexico, where positive cases for* T. gondii* DNA were found through nested PCR.

**Figure 2:**
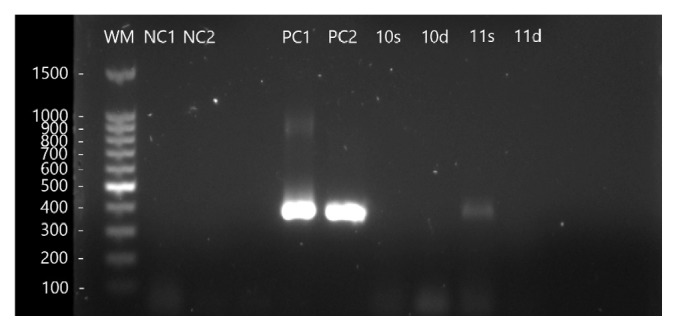
Agarose gel 1.5%. The well 11s (superficial) shows a positive amplification for* T. gondii*. WM: weight marker, NC1: negative control one, NC2: negative control two, PC1: positive control one (1x10^2^ tachyzoites/mL), PC2: positive control two (1x10^3^ tachyzoites/mL).

Presence of* T. gondii* DNA was detected in 11.8% (8/68) of all the evaluated sandboxes (Figure 1). Of the positive samples, 4 were from the superficial region (50%) and 4 from the deep region (50%) (Figure 2). All samples were negative for* T. canis *and* A. caninum.*

## 4. Discussion

The results demonstrate the presence of* T. gondii* in the evaluated sand samples, which represents an important risk factor due to the quantity of people that visit those recreational sites. Results are similar to those reported by Wang et al. in China where they reported that 34 out of 268 (12.69%) soil samples were* T. gondii* positive [10], but lower than those reported by Gotteland et al. [11] in France where 71 out of 243 (29.2%) samples were positive; the difference is that this French village is mostly inhabited and cat populations concentrate which could explain the greater number of positive samples. In the studied region, the diversity of people that visit these places has been reported; from the interviewed people, it was found that 47% of adults visit the parks, 77.8% of interviewed teenagers visit the parks, and 71% of interviewed children also visit the parks [37]. Children represent the principal group at risk for acquiring the infection because of their behavior in playgrounds. In Mexico, seroprevalence of infection has been reported in this age group, with results of 10.4% in children <5 years old, 26.2% in 5-9-year-olds, and 28% in 10-14-year-olds [38]; therefore, it is clear that people are getting infected from an early age and the contact with contaminated soil could be one of the sources of infection, especially in children [39]. Toxoplasmosis is an infection that can cause severe lesions and even death in susceptible groups like people with AIDS and pregnant woman and their products of conception [40]. This infection is considered endemic in the study region, where prevalence of 20-90% has been reported in different population groups [28, 41]. Also, prevalence of* T. gondii* infection in people with AIDS and women with abortions has been reported as 47 and 59%, respectively [42–44], reiterating the impact that this parasite is represented in endemic regions.

The study region has favorable environmental conditions for the viability of* T. gondii* oocysts which is characterized by high temperatures (average: 23.5-30.7°C) and high humidity [32]; under this conditions, oocyst can survive for up to 21 months [7] which highlights its importance as a source of infection for people who come into contact with the infective form. In the same way, in this region, there is a high number of freely roaming cats, which are responsible for the environmental contamination. Cats can excrete up to 10 million oocysts a day and they do it for 7 to 20 days after the primary infection [6], to later develop immunity and stop excreting them. However, experimental studies have shown that they can reeliminate oocysts after a secondary infection [45]. In this region, a total of 433 public parks are present, occupying a total area of 2,329 860 m^2^ (2.32 km^2^) [46]. Some of the characteristics shared by the studied parks are the absence of barriers that prevent access to playgrounds (site of indiscriminate defecation) and the presence of stray animals (dogs and cats) in the parks, which means that any of these parks could be a potential site of defecation for these animals, especially cats, which have the characteristic of burying their feces in particulate and shallow materials such as sand and soil; it has been observed that they develop this habit even without having seen their mother do it [47]; therefore, it is clear that the sand areas of the parks are an ideal place for them to carry out this activity. In addition, in this region, there are no obligatory programs to control the overpopulation of stray cats, which contributes significantly to the maintenance of the contamination problem. Nevertheless, there are other strategies that could reduce this contamination; one of them would be to replace the sand pits of the parks by other materials like rubber floor, which softens falls and is antiskid and highly resistant to water making it ideal for outdoors, which would also prevent cats from excreting their feces in these areas. It is also important to educate the population, especially children, in the implementation of hygiene measures such as the constant washing of the hands after attending these recreational areas and taking care of their behavior to avoid the accidental ingestion of sand. The spatial distribution of the positive samples could be explained by the cat density which is correlated with human population density and food abundance [48]; the city of Merida has an average human population density of 38.02 per km^2^; nevertheless, the highest density is reported in the south followed by the north part of the city, while the west and the east have lower densities [49].

However, it is worth mentioning that all the evaluated sand samples were negative for the presence of* T. canis* and* A. caninum*. This could be due to the behavior of dogs, which unlike cats, seem to have no predilection for a particular type of surface [50]. Despite the negativity in the samples, in the region of study, there has been reported a seroprevalence of* T. canis* of 29.2% in the human population [28]. On the other hand, there are no available reports of* A. caninum* in the human population. However, a 10% positivity was recently reported for* A. caninum* and 1% for* T. canis* in stool samples collected in public parks in the same study region [51]. In addition to* T. gondii*,* T. canis,* and* A. caninum*, the presence of cats and stray dogs circling around public parks represents a source of infection of various diseases such as salmonellosis, campylobacteriosis, leptospirosis, brucellosis, ehrlichiosis, cryptosporidiosis, giardiasis, and leishmaniasis [1, 2]. According to the Center for Disease Control and Prevention, toxoplasmosis and toxocariasis are infections considered as neglected diseases, because they are given little attention in their surveillance, prevention, and treatment, although they are affecting a large number of people with severe consequences in their health [52].

## 5. Conclusion

This is the first report of the presence of* T. gondii* DNA in playground sandboxes samples of the parks of the city of Merida, Yucatan, Mexico, which could represent an important source of infection for the people who visit these areas, especially for children. Control measures should be implemented to reduce the risk of infection in playground areas from parks located in Neotropical areas in Mexico, where the infection caused by* T. gondii* is not considered relevant.

## Data Availability

The data used to support the findings of this study are available from the corresponding author upon request.
